# Impact of TBI on AD‐Associated Proteins in Neuron‐Derived Small Extracellular Vesicles from the Vietnam Era Twin Study of Aging

**DOI:** 10.1002/alz.091711

**Published:** 2025-01-09

**Authors:** Michaela Cullum‐Doyle, Matthew S. Panizzon, Victoria Risbrough, Sonal Sukreet, Haoyi Yang, Jeremy A. Elman, Carol E. Franz, William S. Kremen, Robert A. Rissman

**Affiliations:** ^1^ University of California, San Diego, La Jolla, CA USA; ^2^ Veterans Affairs San Diego Healthcare System, La Jolla, CA USA; ^3^ Alzheimer's Therapeutic Research Institute, University of Southern California, San Diego, CA USA

## Abstract

**Background:**

Alzheimer's Disease (AD) and traumatic brain injuries (TBI) are frequently associated in medical literature, with a significant prevalence of TBI history observed among individuals diagnosed with AD. Our investigation focuses on this intersection, explicitly examining the risk of AD in individuals with a history of TBI. While current targets in cerebrospinal fluid and plasma can effectively detect acute TBI, the challenge lies in identifying biosignatures associated with TBI long after injury. Neuron‐derived small extracellular vesicles (NEVs) from plasma offer potential biomarkers linked to neuronal dysfunction. Prior research has demonstrated that the AD‐associated protein amyloid‐beta (Aβ) is sequestered in NEVs, making them more sensitive biomarkers than free proteins in native plasma. To evaluate the effectiveness of NEV cargo proteins in predicting TBI exposure, we used plasma samples from the third wave of the Vietnam Era Twin Study of Aging (VETSA3). We hypothesized that previous exposure to TBI is associated with abnormalities in AD‐related proteins in circulating NEVs.

**Method:**

Participants in the VETSA3 study, comprising a community‐based sample of male twins aged 61‐73 (average 68), self‐reported a 40% history of mild to moderate TBI. We precipitated NEVs from biobanked VETSA3 plasma samples and enriched them for the neuronal‐specific protein L1CAM using magnetic immunocapture and fluorescence‐activated cell sorting. Following the International Society of Extracellular Vesicles’ guidelines, we characterized NEVs for size, integrity, and homogeneity using NanoSight and ELISA. We quantified AD neuropathogenic proteins Aβ40–42, tau, p‐tau, and Nf‐L in NEVs using SIMOA assays. Our analyses explored the influence of TBI characteristics (number, age at occurrence, severity) on NEV cargo compared to non‐TBI individuals, assessing their utility as potential biomarkers.

**Result:**

NEVs derived from VETSA3 plasma demonstrated similar size distributions and shape characteristics required for cargo analysis. The outcomes of the ELISA validated the presence of the NEV marker protein Flotillin‐1. A significant variation was observed in several neuropathogenic proteins, demonstrating the relationship between TBI exposure and AD.

**Conclusion:**

Our findings give insight into TBI as a risk factor for AD and the role of NEV cargo proteins as potential clinical biomarkers of neurodegeneration.

